# Firing-rate models for neurons with a broad repertoire of spiking behaviors

**DOI:** 10.1186/1471-2202-14-S1-P317

**Published:** 2013-07-08

**Authors:** Thomas Heiberg, Birgit Kriener, Tom Tetzlaff, Gaute T Einevoll, Hans E Plesser

**Affiliations:** 1 Dept. of Mathematical Sciences & Technology, Norwegian Univ. Life Sciences, 1432 Aas, Norway; 2 Inst. of Neuroscience and Medicine (INM-6) and Inst. for Advanced Simulation (IAS-6), Jülich Research Center and JARA, Jülich, Germany

## 

Instantaneous firing rates are commonly used to describe either the compound spiking activity of neuron ensembles (population rate) or the trial-averaged response of individual neurons to multiple repetitions of the same stimulus. The dynamics of firing rates is often studied by means of population or firing-rate models. The main motivation for using such models rather than spiking neuron models is to reduce the dimensionality and complexity of the microscopic dynamics to allow analytical tractability, efficient simulation, and intuitive understanding. In most cases, population models have to be treated as purely phenomenological descriptions. Only under simplifying assumptions can they be derived or extracted from the single-neuron dynamics.

Previous studies in this field were mainly restricted to homogeneous populations of simple integrate-and-fire neurons which can, to some extent, mimic the rather stereotypical behaviour of regularly firing neurons (e.g., pyramidal cells in the neocortex). Electrophysiological studies have revealed a large diversity of neuron types with different dynamical behaviours, ranging from regular spiking to bursting cells, from accommodating to non-accommodating cells, from integrators to resonators, from depolarization- to hyperpolarization-activated cells, etc. So far, the effect of this neuron-type diversity on the dynamics of neural populations is unclear.

Here, we investigate the rate-response characteristics for a variety of neuron types in simulation experiments. To this end, we extend previous work on simple integrate-and-fire neurons [1,2] to the multi-timescale adaptive threshold (MAT) [3] and the Izhikevich model [4]. The model neurons are probed by spike trains modeled as realizations of inhomogeneous Poisson or Gamma point processes with sinusoidally modulated intensity. Average rates, modulation amplitudes and phases of the period-averaged spike responses are measured for a broad range of stimulus, neuron and synapse parameters. The stationary and nonstationary response properties are parameterized by means of nonlinear activation functions and linear filter kernels, respectively, serving as ingredients for simple linear-nonlinear firing rate models. We show that linear-nonlinear rate models can accurately predict the population responses to novel test stimuli for a broad range of parameters and neuron types.
